# Shear Wave Elastography for Carotid Artery Stiffness: Ready for Prime Time?

**DOI:** 10.3390/diagnostics15030303

**Published:** 2025-01-27

**Authors:** Dimitrios Kavvadas, Vasileios Rafailidis, Sasan Partovi, Thomas Tegos, Zoi Kallia, Panagiotis Savvoulidis, Theodora Papamitsou, Panos Prassopoulos

**Affiliations:** 1 Department of Clinical Radiology, AHEPA University Hospital, Aristotle University of Thessaloniki, 54636 Thessaloniki, Greece; billraf@hotmail.com (V.R.); pprasopo@auth.gr (P.P.); 2 Interventional Radiology, Cleveland Clinic Main Campus, Cleveland, OH 44195, USA; sxp509@case.edu; 3 1st Department of Neurology, AHEPA University Hospital, Medical School, Aristotle University, 54453 Thessaloniki, Greece; thomtegos@gmail.com (T.T.); zoi.kallia22@gmail.com (Z.K.); 4 Royal Brompton & Harefield Hospitals, Part of Guy’s and St Thomas’ NHS Foundation Trust, London SW3 6NP, UK; 5 Histology and Embryology Laboratory, Faculty of Health Sciences, Medical School, Aristotle University of Thessaloniki, 54124 Thessaloniki, Greece; thpapami@auth.gr

**Keywords:** shear wave elastography, carotid stiffness, atherosclerosis, ultrasound

## Abstract

Carotid artery stiffness is associated with aging and atherosclerotic disease, leading to cerebrovascular events. Shear Wave Elastography (SWE) is a novel ultrasound technique offering a direct, quantitative assessment of the arterial wall elasticity. The aim of this study is to validate the technical feasibility of SWE in measuring carotid stiffness (CS). A literature search was performed across the PubMed and Scopus databases, with keywords including “carotid stiffness”, “Shear Wave Elastography”, “atherosclerosis”, and “vascular elasticity”. The findings reveal the potential of SWE in quantifying carotid Intima–Media Complex (IMC) stiffness, with implications for the early diagnosis of vascular disease, aiding in clinical decision making and prognostic assessment. Based on the findings of the literature search, a small pilot study was conducted involving 10 participants, using the Philips EPIQ Elite system for the SWE measurements. The technical analysis revealed optimizing the region of interest (ROI) size, probe positioning, and cine-loop analysis as crucial factors for obtaining accurate results. The results of the literature review and small pilot study demonstrate the potential of SWE as a non-invasive method for assessing carotid stiffness. Certain technical adjustments, such as smaller ROIs and careful probe placement, improved the accuracy and repeatability of carotid SWE measurements. Further studies are needed to assess and standardize carotid SWE across larger patient populations.

## 1. Introduction

Ultrasound (US) is the initial modality of choice for assessing carotid stiffness, atherosclerosis, and stenotic disease [1,2]. In recent years, there have been evolving technological sonographic approaches for investigating atherosclerotic carotid disease, such as contrast-enhanced US (CEUS) [3], ultra-fast vector flow US, and elastography [2,4,5]. The success of ultrasonography for this common disease entity relies on its widespread availability, accessibility, cost-effectiveness, and constant evolving technological advancements [6,7]. Novel US modalities assess not only the stenotic component of atherosclerotic plaques, but also some of its functional characteristics, such as carotid wall stiffness [6,7]. Characterization beyond stenotic disease can been labeled as “multi-parametric ultrasound” [8].

The carotid Intima–Media Thickness (IMT), Pulse Wave Velocity, and β-stiffness index have been widely studied and are regarded as appropriate tools for the detection of carotid stiffness [7,9,10,11,12,13,14]. These techniques are valuable because they offer insights into vascular health and potential early indicators of atherosclerosis. However, they provide indirect stiffness indexing based on hemodynamic changes, velocities, and thickness associated with atherosclerotic plaques. For instance, IMT measures structural changes in the arterial wall, while PWV assesses the velocity at which pressure waves travel through the arteries. Similarly, the β-stiffness index integrates blood pressure measurements to calculate arterial stiffness [7,9,10,11,12,13,14].

On the other hand, Shear Wave Elastography (SWE) measures the elastic properties of tissues through measuring the propagation speed of shear waves [15,16], thus providing a direct assessment of the elasticity of vascular walls. This propagation speed of shear waves is directly related to tissue stiffness, with increased speeds indicating greater stiffness, while lower speeds suggesting more flexible (less stiff) tissues, allowing for the evaluation of many organs in the human body, including blood vessels [16]. Thus, measuring the Young’s modulus (which is derived from the wave propagation speeds) in the carotid arteries, particularly in the Intima–Media Complex (IMC), may offer direct quantitative information about the local arterial stiffness of the carotid arteries [17,18]. The above characteristics make SWE an interesting modality for the early detection of vascular changes at early stages, given its non-invasive nature, which enables straightforward integration into daily clinical practice [19].

The Intima–Media Complex (IMC) is particularly sensitive to stiffness changes [20]. Research has demonstrated a significant correlation between the IMC thickness and the early development of atheromatous plaque formation. These changes lead to the onset of cerebral small vessel disease (CSVD), and eventually to increased generalized cardiovascular risk [20]. The IMC is therefore regarded as an important imaging biomarker for carotid atherosclerotic disease evaluation [21]. Therefore, it is important to assess the IMC through imaging techniques like SWE to improve the risk stratification and implement preventative strategies.

## 2. Pathophysiology of Vascular Stiffness

The loss of elasticity in the vascular walls has been regarded as the main “culprit” of arterial aging, and is attributed to both the natural progression of the body and to cardiovascular risk factors [22,23,24]. The basic model of increased vascular stiffness is based on the following three components: clinical factors (hypertension, diabetes, renal failure, and atherosclerosis), arterial wall remodeling (changes in the collagen–elastin ratio, vascular calcification, endothelial dysfunction, Intima–Media Thickening, and genetic predispositions), as well as environmental risk factors (smoking, reduced physical activity, and alcohol consumption) [25]. Arterial stiffness has emerged as an independent prognostic indicator of cardiovascular events and mortality [26]. Specifically, it leads to an inability to dampen the changes in blood pressure caused by cardiac function, resulting in increased systolic blood pressure, decreased diastolic pressure, and elevated pulse pressure [27]. Additionally, it burdens the brain’s microcirculation and is therefore associated with microbleeds, cognitive impairment, and stroke [28].

### 2.1. Factors Associated with Carotid Stiffness

Recent ultrasound protocols are focusing on the IMC using SWE to estimate the Young’s modulus (YM) in different populations, aiming to create a dataset for future reference. SWE allows for a more precise evaluation of the IMC’s mechanical characteristics compared to traditional methods and offers an advantage in its ability to detect subtle changes in the IMC’s stiffness [29]. Therefore, studies have focused on several factors that are associated with CF, as measured by SWE. Goksu et al. [30] identified smoking and dyslipidemia as independent factors associated with an increased YM in the IMC. Based on the study results, even one cigarette a day may increase CS, and further research is warranted on the amount and duration of smoking in relation to CS [30,31]. Two additional studies with SWE focused on smoking, alcohol consumption, and a high Body Mass Index (BMI). The findings suggest elevated CS in these groups (increased YM on the IMC) [30]. Similar findings were published by Zhang et al. on diabetic patients with fatty liver disease [29].

Apart from the risk factors, studies have been aimed at demographics as well. Different demographic characteristics seem to increase the possible heterogeneity of CS. It is recommended that future studies include possible CS changes in relation to age, gender, and ethnicity [32,33,34]. Thus far, the β-stiffness index and the CIMT have been widely used in relation to gender and age, because of the natural aging of the arteries, and in relation to possible risk factors (e.g., smoking and cardiovascular comorbidities) [35,36]. Women seem to show a stronger correlation between CSVD and age compared to men due to the cessation of the protective effects of hormones on the arterial vasculature [37]. The literature suggests that the YM of healthy individuals with an average age between 40 and 50 years fluctuates around 50 kPa [33,34,38].

Another significant independent risk factor for increased CS is the accumulation of fat on the pericarotid space [39,40]. There is evidence that pericarotid fat is associated with leukoencephalopathies, CSVDs, and brain infarcts. Correlations of pericarotid fat were also found with age, hypertension, and hyperlipidemia [41]. An increased pericarotid fat density was associated with high-risk vulnerable atherosclerotic carotid plaques, suggesting that increased inflammation of perivascular fat is linked to carotid artery stenosis [42]. Increased fat density surrounding the internal carotid arteries (ICAs) is associated with the risk of future stroke or transient ischemic attacks, suggesting a bidirectional relationship, where a higher pericarotid fat density may not only result from but also contribute to vascular pathology [42]. The amount of pericarotid adipose tissue was also associated with carotid artery atherosclerotic diseases and characteristics of chronic atherosclerotic lesions, namely, the presence of carotid plaques, increased IMT, and the area of the largest plaque [39,40].

The degree of carotid stiffness (measured using flow techniques and/or the calculation of the β-stiffness index) is a strong risk factor for atherosclerosis and CSVD. These conditions are major contributors to adverse cerebrovascular outcomes, including stroke, highlighting the critical importance of early detection and intervention [36,43]. Despite the well-documented relationship between carotid stiffness and cerebrovascular risk, there are very limited studies regarding the application of SWE on the IMC as an early indicator of carotid stiffness. SWE could emerge as a useful US modality for improving cardiovascular and cerebrovascular risk assessments in clinical practice.

### 2.2. From Carotid Stiffness to Cerebral Small Vessel Disease and Plaques

Several studies have established a link between carotid atherosclerosis, microangiopathy, and cerebral blood flow disorders, resulting in the development of chronic ischemic lesions and an increased risk of stroke [44]. In patients who experienced acute ischemic stroke due to small artery occlusion in the brain, with associated neurological symptoms, increased [45] and reduced wall shear stress (WSS) were found in the carotid arteries [46]. As these factors are directly associated with the development of atherosclerosis, there could be a relationship between the risk of neurological symptomatology due to arterial atheromatosis and CS at early disease stages. In a study investigating the relationship between the aging of the brain and carotid changes, it was found that a low end-diastolic velocity, increased resistance, PWV, and pulsatility of the carotid arteries were associated with signs of cerebral small vessel disease and brain atrophy [47,48,49]. Rundeck et al. reported a positive a correlation between the diameter of the common carotid artery and the Fazekas score, regardless of the IMT and the presence of atheromatous plaques [50]. Jackson et al. highlighted the correlation between strain elastography in carotid atheromatous plaques and cognitive function related to transient ischemic attacks (TIAs). Thus, subclinical carotid atherosclerosis may be a modifiable risk factor associated with cognitive decline in the middle- and older-age population [51,52].

The ability of SWE to quantify the elasticity of the IMC and atheromatous plaques is useful for predicting microangiopathy, new plaque formation, or the risk of rupture of existing plaques. The negative correlation between intraplaque neovascularization and plaque elasticity is suggestive of the risk associated with plaques that have high levels of intraplaque neovascularization, as they are more prone to rupture [53,54,55]. SWE can also visualize the thickness of the plaques’ fibrous cap, which is important for assessing carotid plaque stability, especially in hypoechoic plaques [2,56,57,58]. More specifically, stable plaques have a YM of approximately 52–88 kPa, while unstable plaques (in patients with a history of stroke) have lower YM values (30–62 kPa) [53,54,59,60,61]. Further studies exploring the application of SWE in larger populations could enhance its possible diagnostic value and expand its role in preventing cerebrovascular events.

## 3. Technical Guidelines

Research on SWE on CS proves the dire need for a proper technical guide to be integrated into daily clinical practice for accurate SWE measurements [17,19,32,33,34,43,56,60,62,63,64]. The literature recommends conducting pilot studies to verify the repeatability of measurements and the variability of values, with the goal of identifying a specific pattern of increased validity for adoption in both research and clinical practice.

Shear Wave Elastography studies on carotid stiffness are controversial regarding the following approaches:

- Measurements on the posterior or anterior carotid wall: One study recommends the anterior wall [34], while another finds it favorable that the posterior part adheres stably to the muscles [32]; however, the distance of the posterior wall from the probe causes the reduced repeatability of the results [34]. On the other hand, it has been suggested that the anterior wall may have errors due to the direct contact with the jugular vein, resulting in mobility causing artefacts [32,34].
- Possible differences in CS between the right and left side: Research suggests that side-related differences may exist, potentially due to anatomical or hemodynamic factors [32]. On the other hand, a pilot study assessing the SWE imaging of CCAs found no significant difference in the YM between the left and right sides [62]. It is essential to consider that individual characteristics can influence results.
- Internal variability of measurements (intra-/inter-observer variability): To evaluate the intra-observer reproducibility of the YM, it is proposed that the same measurements should be repeated at different time points within a time frame of one week minimum to several months. Also, a second independent examiner should repeat the measurements twice to assess the inter-observer reproducibility of the results. Both observers must be blinded to each other’s findings [32,61]. These measurements will also assist with the possible differences related to the carotids’ depth and location.
- Measurements during systolic and diastolic cardiac phases: A published case reports a difference of almost 60% between the two phases, with the systolic YM to be up to 130 kPa [65]. It seems that the shear wave speed in the carotid artery increases with blood pressure throughout the cardiac cycle, resulting in a higher stiffness during the systole compared to diastole.

### 3.1. Technical Recommendations

The following suggestions are based on the published research on measuring CS with SWE:

- Dual Mode, with a B-Mode image on the left and an elastography image on the right [63]. Shang et al. also suggests split screen, with a real-time gray-scale and concurrent SWE image [58]. Dual mode provides simultaneous data in anatomy and elastography, allowing for the precise placement of the region of interest (ROI), while ensuring the proper alignment of SWE. Without dual-mode imaging, the ROI placement may be less accurate.
- The scanner SWE settings should be standardized to optimize acquisitions based on the initial clinical experience and technician advice, minimizing the variability due to operator or equipment differences. More specifically, a depth of image at 3 cm; acoustic power (maximum); smoothing (mid-range setting of 6); persistence (off); gain (65–70%); SWE option (penetration) [58,60].
- The shear waves should be created using three successive pushes of 10 s at three depths, 5 mm apart along the centerline, of a manually positioned ROI [66]. This ensures the sufficient propagation of the shear waves and allows for the assessment of tissue elasticity at varying depths.
- A 2D color map of the shear wave velocities (SWVs) should be acquired, as it aids with the identification of homogeneous areas where the measurements are the most reliable. ROIs should be placed where the map shows a homogenous SWE distribution [56].
- Images should be magnified for better ROI acquisition (suggested at 200%), as poor visibility without magnification may lead to errors in the stiffness measurements due to imprecise ROI placement [66].
- Breath-holding minimizes motion artifacts caused by respiratory movement; therefore, participants should hold their breath for 3 s to ensure image stability [66].
- The ultrasound probe should be perpendicular to the skin, and mild pressure should be applied for 10 s to ensure uniform wave propagation and to avoid the distortion of soft tissues [32,58].
- A frame rate of 1 Hz with 10 frames is suggested, as it ensures accurate elastography data during a stable acquisition phase. The initial frames should be discarded to ensure equipment stabilization. The selection of specific frames minimizes the transient noise [60].
- From the acquired cine-loops, the first two SWE frames should be discarded to allow the SWE acquisition to stabilize [60,62]. Another suggestion is that, from the 10 frames, the 3 first frames and 3 last frames should be discarded, and 4 frames should be acquired for the measurements [58]. Shorter loops or insufficient frame analysis may lead to errors.
- The cine-loops should last for 10 s in a longitudinal view of the vessel (common carotid artery), approximately 2 cm from the carotid bulb, and four to five consecutive frames should be analyzed to calculate the average Young’s modulus from these frames. This approach should yield 40 Young’s modulus values per participant, that is, four ROIs times five frames times two sides [58,60,62,63].
  ○ In one case [62], the four ROIs include two on the anterior and two on the posterior carotid walls for the five frames. The rectangular region of interest (box) should be 2 cm in length and 1.5 cm depth if applied on both walls [32].
  ○ In case of a one-sided selection, the number of ROIs should be recalculated accordingly [32].
  ○ Regarding the study position of the Intima–Media Complex, other studies measured three ROIs, with one ROI in each area of interest, e.g., one ROI in the common carotid artery, one ROI in the internal carotid artery, and one ROI in the carotid bulb (totaling six ROIs for both the left and right sides) [43,67].
- For the ROI size, it is suggested that, for a CIMT on the CCA of 0.6 mm, an ROI of 2 mm should be used. The idea behind this is that large ROIs create heterogeneity in the measurement, while very small ROIs may introduce operator error [34,60]. Researchers may also explore in the future the use of 1 mm ROI sizes [67].

### 3.2. Assessing Carotid Plaques with Shear Wave Elastography

Regarding plaques, SWE appears to demonstrate very good reproducibility with minor differences between ultrasound machines [64]. In the case of detecting an atherosclerotic plaque, both longitudinal and transverse sections should be taken, and the following criteria should be examined (FMB-UNESP) [68]: appropriate longitudinal ultrasound beam angle, sufficient differentiation of the Intima–Media Complex, the delineation of the proximal and outer layers (tunica adventitia) (kPa > 7.1), the delineation of the vascular lumen (kPa < 7.1), the delineation of the atherosclerotic plaque, and the absence of significant changes in the plaque over time.

### 3.3. The Challenge with the Cardiac Cycle

Regarding the cardiac cycle, it is suggested that images should be taken in accordance with the diastolic phase, as this phase constitutes the largest part of the cardiac cycle, resulting in the retraction of the vascular wall to its initial morphology after the passage of the pulse wave. In the diastolic phase, more accurate information regarding the increase in arterial wall stiffness can be obtained secondary to the longer available processing time for the shear waves [63,69]. Measurement during the diastolic phase is feasible without electrocardiogram gating, since it is possible to differentiate the measurements based on the cardiac cycle using images in dual mode and cine-loops [63].

### 3.4. Measurements’ Reproducibility

In several studies, the intra-observer reproducibility for SWE has been reported as fair to good, with coefficients ranging from 0.46 to 0.71 [58,60,64]. The inter-observer coefficient of variation was shown to be between 0.16 and 0.22 for vessels and 0.19 for carotid plaques [64], while the intra-observer coefficient of variation turned out to be 0.14 (which ranged from 0.07 to 0.22) [58,60,64]. Another study reported a coefficient variation that ranged from 1% to 135%, with a mean of 26% [34]. This means that the variation in the YM measurements among the frames showed significant diversity, and the average variability was 26%. In the study by [34], the authors compared a total of 1016 ROIs for 127 participants on the left–right/anterior–posterior/–distal sites of CCA and found no significant differences, apart from anterior–posterior wall comparisons on both sides (proximal and distal CCA) and the right anterior proximal wall [34]. Interestingly, their findings conclude that the left CCA measurements were more variable compared to their right-sided counterparts (14%), with more conspicuous differences on the posterior wall [34]. The above indicates that there is a reasonable consistency among the measurements made by the same observer, but there is some measurement variation among the different frames in these studies [32,54,58,60,61].

## 4. Small Pilot Study

To confirm the feasibility of the published technical instructions, a small IRB-approved pilot study was conducted at the Medical School of the Aristotle University of Thessaloniki from 15 November to 30 November 2024 (the Aristotle University of Thessaloniki, Medical School Bioethics Committee Number: 6/2024, date: 13 October 2024). The Philips EPIQ Elite system with the eL18-4 ultra-broadband linear array transducer with PureWave crystal technology was utilized. An elastography protocol for measuring CS was developed based on literature guidelines for the SWE measurements in this pilot study [17,19,32,33,34,43,56,60,62,63,64]. For measuring the arterial pressure, a certified digital blood pressure monitor device was used.

**Figure 1:**
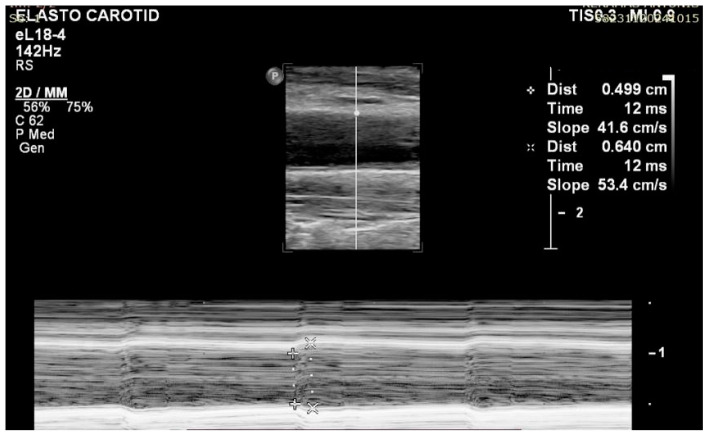
M-Mode image for calculating the β-stiffness formula. Systolic and diastolic diameters were measured to derive the arterial stiffness based on β-stiffness index.

Ten healthy volunteers who gave informed consent were asked to relax for 5 min prior to the examination. The examination was performed by an experienced radiologist with years of experience in imaging on a Philips EPIQ Elite Ultrasound System (Philips Healthcare, Andover, MA, USA). The SWE analysis was conducted independently by two examiners. Participants were placed supine with their head in neutral position. Measurements were made in the distal CCA. Three BP measurements were taken in supine position before the SWE examination (two on the left and one on the right hand). Means were extracted from the two left-sided measurements and, through an M-Mode imaging, the characteristics for calculating the β-stiffness index were determined. SBP and DBP were added in the formula based on the average value from the two left-side measurements. Systolic and diastolic diameters were calculated with five different M-Mode images on the left CCA (Intima-to-Intima distance on each phase), and the average was added to the formula for each participant. The β-stiffness index was calculated with the following formula:

$$\beta \;stiffness\;index=\frac{ln\;ln\;(\frac{SBP}{DBP})}{\left(\frac{Ds-Dd}{Dd}\right)}$$

where *SBP* and *DBP* stand for the systolic and diastolic blood pressure, respectively; *Ds* equals the systolic diameter and *Dd* equals the diastolic diameter (Figure 1).

**Figure 2:**
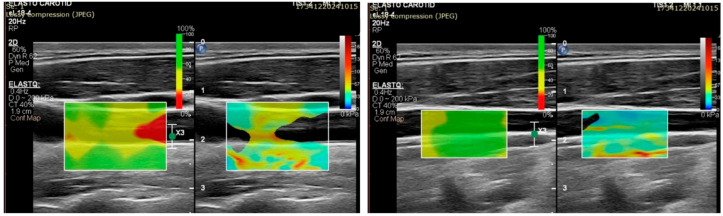
Comparison of the confidence maps during the diastolic phase when the ROI box was positioned on the anterior and posterior walls versus the sole ROI box placement at the posterior carotid wall. Confidence maps are shown on the right of each figure. “Green” areas suggest a safe measurement. An ROI box placement on the right figure enhanced the SWE measurement reliability by reducing the motion artifacts.

**Figure 3:**
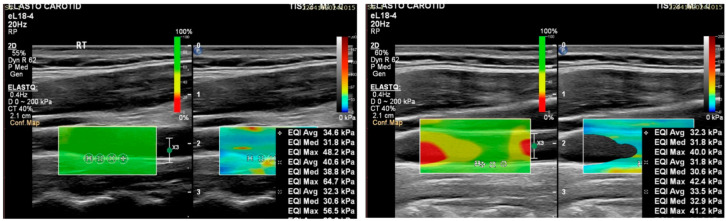
Comparison of ROIs’ diameter during the diastolic phase. Measurements show improved precision with the 1 mm ROI (right figure) compared to the 2 mm ROI (left figure). Confidence maps are shown on the right of each figure. “Green” areas suggest a safe measurement. The ROIs were placed on the IMC and the 1 mm ROIs presented a more accurate set of measurements.

A split screen with a real-time gray scale and SWE was applied in this pilot study based on suggestions in the literature. The focal distance, time gain compensation, and gain were standardized during the SWE measurements to obtain the maximum morphological characteristics of the sites (Figure 2 and Figure 3). The depth of the image was set at 2 to 3 cm, with acoustic power (maximum), smoothing (mid-range setting of 6), persistence (off), and gain (55–65%). A 2D color map of SWV was acquired, and the ROIs were placed at homogenous SWE distributions. The participants were instructed to hold their breath and stay still so to ensure adequate image stability. The probe was placed perpendicular to the skin, which was coated with plenty of ultrasound gel, and mild pressure was applied for 10 s.

From the acquired cine-loops (ten frames), the first two and last two were discarded, and six frames were acquired for measurement purposes. From the six consecutive frames, measurements were taken from those at the diastolic phase according to the B-Mode image. The average Young’s modulus was determined from the frames at diastolic phase (2–3 frames). To address questions arising during previously published studies, the CS on both sides was measured (right and left) and attention was drawn to the posterior carotid wall (three ROIs times two to three frames: approximately six to nine ROIs per cine-loop and side (left/right)) on the posterior carotid wall of each participant. CS was measured by two independent observers to enable a preliminary assessment of the inter-observer variability.

Initially, a significant error on the confidence map was noted when the ROI box was positioned to cover both the anterior and posterior walls, possibly due to the movement of both walls and nearby structures. An increased stability of the confidence map was achieved when the ROI box was positioned solely on the posterior wall, which was likely related to the reduced mobility (Figure 2).

The ROI size of 1 mm instead of 2 mm was feasible in this pilot study, secondary to the clear image of the IMC acquired with the proper magnifications. ROIs of 1 mm diameter are advantageous secondary to the reduced risk of measuring nearby structures or receiving signal from blood flow (Figure 3).

**Figure 4:**
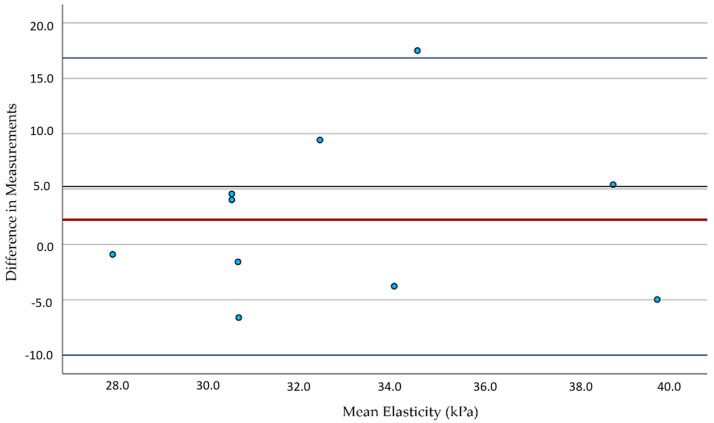
Bland–Altman plots for the left CCA measurements by two independent observers. The red line illustrates the agreement between the two independent examiners, showing mild inter-observer variability. The blue lines highlight the intra-observer variability. The blue dots represent the individual data points for the elasticity differences, while the dark gray line represents the mean difference across all measurements.

**Figure 5:**
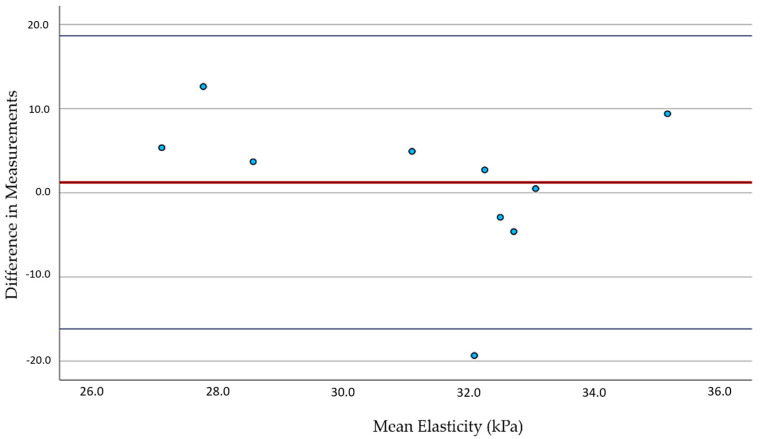
Bland–Altman plots for the right CCA measurements by two independent observers. The red line illustrates the agreement between two independent examiners, showing no significant difference. The blue lines highlight the intra-observer variability.

The results were consistent with the literature for healthy participants (Table 1). For smokers, our pilot revealed significant differences only related to the left CCA measurements (Table 1). The analysis showed a normal distribution of values (Shapiro–Wilk Test). The Bland–Altman plots for the left- and right-sided carotid arteries were utilized to measure the inter-observer differences between the two examiners. The left CCA revealed a mild inter-observer variability between the examiners (red line, Figure 4) and, compared to the right CCA, a similar intra-observer variability (blue lines, Figure 4 and Figure 5). Regarding the right CCA, there was no significant difference between the two observers (red line, Figure 5).

Based on the literature, gender may affect the CS measurements using SWE. In this pilot, the mean age was 26 (5.5) and 55.3 (6.3) for non-smokers and smokers, respectively. Our three smokers were men and, among the seven non-smokers, three women were included. All of the participants showed a normal BMI (18.5 to 25). It needs to be emphasized that these results are related to a pilot study using the Philips EPIQ Elite machine to check the feasibility of measuring CS with SWE. Therefore, a clinical conclusion cannot be drawn at this point and, due to the limited number of participants, no further sub-analyses about gender or age were performed.

Regarding the life-time smokers participating in the study, the β-stiffness turned out to be elevated, though still in the normal range for their respective age group. The IMT in the smokers was also elevated compared to the non-smokers, but still not above 1 mm. The left CCA measurements showed a mild inter-observer variability due to the reading differences between the two independent examiners, while the imaging was standardized. The right CCA presented agreement with increased stiffness in smokers, though no significant difference was found between the smokers and non-smokers in this small pilot study. It needs to be taken into consideration that the increased values in the β-stiffness and IMT of the smokers’ groups could be biased by age. Of note, carotid atherosclerotic plaques were not detected in either group.

## 5. Conclusions and Future Perspectives

This paper provides a comprehensive technical guide for assessing carotid artery stiffness with SWE as a non-invasive approach for direct arterial elasticity measurements. The associated pilot study revealed the difficulty of performing SWE for measuring CS in daily clinical practice. Minor technical interventions were performed during the pilot study, such as the selection of ROIs with a diameter at 1 mm and applying the confidence box position. Repeatability was encountered in this pilot study, and the obtained results on the CS measurement using SWE were close to previously reported values. The observed intra-observer variability in this small pilot study turned out to be good. The inter-observer variability was less optimal, which is in line with prior studies. The challenges encountered during the pilot study were related to the constant movement of the carotid walls and the blood flow. SWE has the potential to serve as a valuable sonographic modality for real-time stiffness evaluation; therefore, the continued development on atherosclerotic disease is worthwhile, and pursuing larger studies in different patient populations is warranted.

CS measurements using SWE, and plaque imaging in general, are based on gradual changes due to the deposition of lipids in the Intima. In the future, the radiomics evaluation of carotid plaques may be feasible by integrating CS as a biomarker based on SWE measurements [70,71]. Images of the carotid artery walls with different ROIs should be acquired on several segments of the vessel. After segmentation, quantitative features could be extracted from the segmented ROIs based on the stiffness variations and texture within the arterial wall. Radiomic features could be used to develop a model predicting vascular conditions based on increased vessel wall stiffness, indicating early microangiopathy and atherosclerotic disease before plaque formation occurs [72]. The movement of carotid walls and the effect of blood pressure fluctuations could be addressed through advanced motion compensation algorithms and improved software. Also, advancements in instrumentation, such as faster measurement times, improved probe ergonomics, and higher resolution imaging, could significantly reduce the technical difficulties.

This article, along with the integrated small pilot study, may serve as a basis for larger projects assessing CS using SWE. The review provides evidence of the significance of integrating modern modalities like SWE into clinical practice. However, SWE is a difficult technique to perform on vessels, as confirmed by the pilot. There were several technical obstacles during the implementation of the SWE protocol in the pilot study. Nevertheless, it should be tested further as an evolving ultrasound modality in individuals with increased risk factors for carotid atherosclerotic disease.

## Figures and Tables

**Table 1:** Summary of the results of measuring CS with SWE in healthy volunteers.

Smokers/Mean (SD)	β-Stiffness Index	IMT (mm)	SBP	DBP	Left_CCA1 (kPa)	Left_CCA2 (kPa)	Right_CCA1 (kPa)	Right_CCA2 (kPa)
**NO** **(*n* = 7)**	2.56 (0.53)	0.46 (0.03)	118.1 (7.7)	72.3 (7.7)	33.5 (3.3)	27.9 (4.0)	32.1 (5.4)	30.2 (3.6)
**YES** **(*n* = 3)**	4.09 (1.41)	0.73 (0.15)	140.7 (8.1)	84.7 (4.5)	28.0 (4.8)	37.0 (4.2)	38.5 (2.5)	35.2 (7.7)
***p*-Values**	0.029 ^1^	0.001 ^1^	0.003 ^1^	0.034 ^1^	0.048 ^1^	0.012 ^1^	0.092	0.182

^1^ Student’s *t*-Test, *p* < 0.05. CCA1: 1st examiner; CCA2: 2nd examiner; SBP: systolic blood pressure; DBP: diastolic blood pressure, IMT: intima media thickness.

## Data Availability

No new data were created or analyzed in this study.

## Abbreviations

The following abbreviations are used in this manuscript:

SWE	Shear Wave Elastography
IMC	Intima–Media Complex
CS	Carotid Stiffness
CEUS	Contrast-Enhanced US
IMT	Carotid Intima–Media Thickness
CSVD	Cerebral Small Vessel Disease
YM	Young’s Modulus
BMI	Body Mass Index
ICAs	Internal Carotid Arteries
TIAs	Transient Ischemic Attacks
CCA	Common Carotid Arteries
SBP	Systolic Blood Pressure
DBP	Diastolic Blood Pressure
